# Correction of LAMP3-associated salivary gland hypofunction by aquaporin gene therapy

**DOI:** 10.1038/s41598-022-21374-2

**Published:** 2022-11-03

**Authors:** Hiroyuki Nakamura, Tsutomu Tanaka, Changyu Zheng, Sandra A. Afione, Blake M. Warner, Masayuki Noguchi, Tatsuya Atsumi, John A. Chiorini

**Affiliations:** 1 Adeno-Associated Virus Biology Section, National Institute of Dental and Craniofacial Research, National Institutes of Health, 10 Center Drive, Bethesda, MD 20892 USA; 2 Salivary Disorder Unit, National Institute of Dental and Craniofacial Research, National Institutes of Health, Bethesda, MD USA; 3 Division of Cancer Biology, Institute for Genetic Medicine, Hokkaido University, Sapporo, Japan; 4 Department of Rheumatology, Endocrinology and Nephrology, Faculty of Medicine, Hokkaido University, Sapporo, Japan

**Keywords:** Rheumatic diseases, Cell biology, Membrane trafficking, Endocytosis, Lysosomes, Molecular medicine, Viral vectors

## Abstract

Sjögren’s disease (SjD) is a chronic autoimmune sialadenitis resulting in salivary gland hypofunction with dry mouth symptom. Previous studies showed that lysosome-associated membrane protein 3 (LAMP3) overexpression is involved in the development of salivary gland hypofunction associated with SjD. However, the molecular mechanisms are still unclear, and no effective treatment exists to reverse gland function in SjD. Analysis on salivary gland samples from SjD patients showed that salivary gland hypofunction was associated with decreased expression of sodium–potassium-chloride cotransporter-1 (NKCC1) and aquaporin 5 (AQP5), which are membrane proteins involved in salivation. Further studies revealed that LAMP3 overexpression decreased their expression levels by promoting endolysosomal degradation. Additionally, we found that LAMP3 overexpression enhanced gene transfer by increasing internalization of adeno-associated virus serotype 2 (AAV2) via the promoted endolysosomal pathway. Retrograde cannulation of AAV2 vectors encoding *AQP1* gene (AAV2-AQP1) into salivary glands induced glandular AQP1 expression sufficient to restore salivary flow in LAMP3-overexpressing mice. LAMP3 could play a critical role in the development of salivary gland hypofunction in SjD by promoting endolysosomal degradation of NKCC1 and AQP5. But it also could enhance AAV2-mediated gene transfer to restore fluid movement through induction of AQP1 expression. These findings suggested that AAV2-AQP1 gene therapy is useful in reversing salivary gland function in SjD patients.

Saliva plays an essential role in our health by initiating the digestive process and controlling oral infections^1^. Sjögren’s disease (SjD) is a chronic disease that leads to secretory hypofunction with dry mouth and/or eye (sicca) symptoms, which impairs patients’ oral and eye health as well as their quality of life. This autoimmune exocrinopathy is characterized by lymphocytic infiltration into the affected glands and the presence of serum autoantibodies, such as anti-Ro/SSA and anti-La/SSB antibodies^2^.

In SjD, which is regarded as an epithelitis, epithelial dysfunction of glands is a prominent feature, which can eventually result in epithelial apoptosis^3^. Recent microarray and confocal immunofluorescence studies have shown that increased epithelial apoptosis in salivary glands of SjD patients compared to healthy glands is associated with increased expression of lysosome-associated membrane protein 3 (LAMP3)^4–6^. In addition, we have previously shown that LAMP3 overexpression decreases expression of plasma membrane proteins involved in salivation, such as sodium–potassium-chloride cotransporter-1 (NKCC1) and aquaporin 5 (AQP5), in salivary gland epithelial cells^6^. Moreover, LAMP3-overexpressing mice develop SjD-like salivary gland hypofunction, whereby the salivary flow rate (SFR) shows a strong positive correlation with NKCC1 and AQP5 expression levels^6^. Although decreased NKCC1 and AQP5 expressions are considered critical for the development of secretory hypofunction in the mouse model, mechanistically, it is unclear how LAMP3 decreases expression of these membrane proteins.

NKCC1 is an ion cotransporter expressed in basolateral membrane of salivary gland epithelial cells and plays a critical role in exocrine gland fluid secretion^7–9^. AQPs are a family of water channels that facilitate the movement of water across plasma membrane and have many key functions including fluid homeostasis and glandular secretion^10,11^. Among the AQPs, AQP5 is expressed in apical membrane of salivary gland epithelial cells, while AQP1 is an archetypical and unregulated water channel that can be expressed on both the apical and basolateral membranes of various cells^11^. Thus, expression of AQP1 in the salivary gland epithelia creates a novel route for fluid secretion in the salivary glands^12^.

Because the pathophysiology driving salivary gland hypofunction in SjD has been poorly understood, only palliative treatment exists for alleviating sicca symptoms in SjD patients^2^. Clarifying the molecular details in LAMP3-associated salivary gland hypofunction is essential to develop a novel therapeutic approach for restoring gland function in SjD.

In the present study, we showed that LAMP3 can promote degradation of NKCC1 and AQP5 through activation of the endolysosomal pathway, resulting in salivary gland hypofunction. This finding suggested that restoration of gland function would require the development of a new pathway instead of NKCC1 and AQP5 to initiate and transit fluid in the salivary glands. One potential therapy could involve gene transfer via adeno-associated virus (AAV) vectors. We found that LAMP3 overexpression can increase efficiency of gene transduction by upregulating endocytosis of AAV serotype 2 (AAV2). The effect of LAMP3 on AAV2 transduction suggested that it could enhance gene transfer and expression. Finally, we observed that AAV2-mediated *AQP1* gene therapy could restore SFR in LAMP3-overexpressing mice. These findings support AAV2-mediated *AQP1* gene therapy to replace decreased NKCC1 and AQP5 expressions as a therapeutic approach in correcting LAMP3-associated salivary gland hypofunction in SjD patients.

## Results

### Decreased NKCC1 and AQP5 expressions are associated with salivary gland hypofunction in SjD patients

We have previously shown that decreased NKCC1 and AQP5 expressions are associated with salivary gland hypofunction in LAMP3-overexpressing mice^6^. To examine whether the same findings are evident for SjD patients, we acquired labial minor salivary gland biopsies from 11 individuals with sicca symptoms (Fig. 1A and Supplementary Table 1). Immunofluorescent staining revealed a significant decrease in NKCC1 and AQP5 expression levels in the glands of SjD patients with decreased SFR (*n* = 5) compared to those of control subjects with normal SFR (*n* = 6) (Fig. 1B). These findings suggested that decreased NKCC1 and AQP5 expressions are associated with salivary gland hypofunction in SjD patients as well.

**Figure 1:**
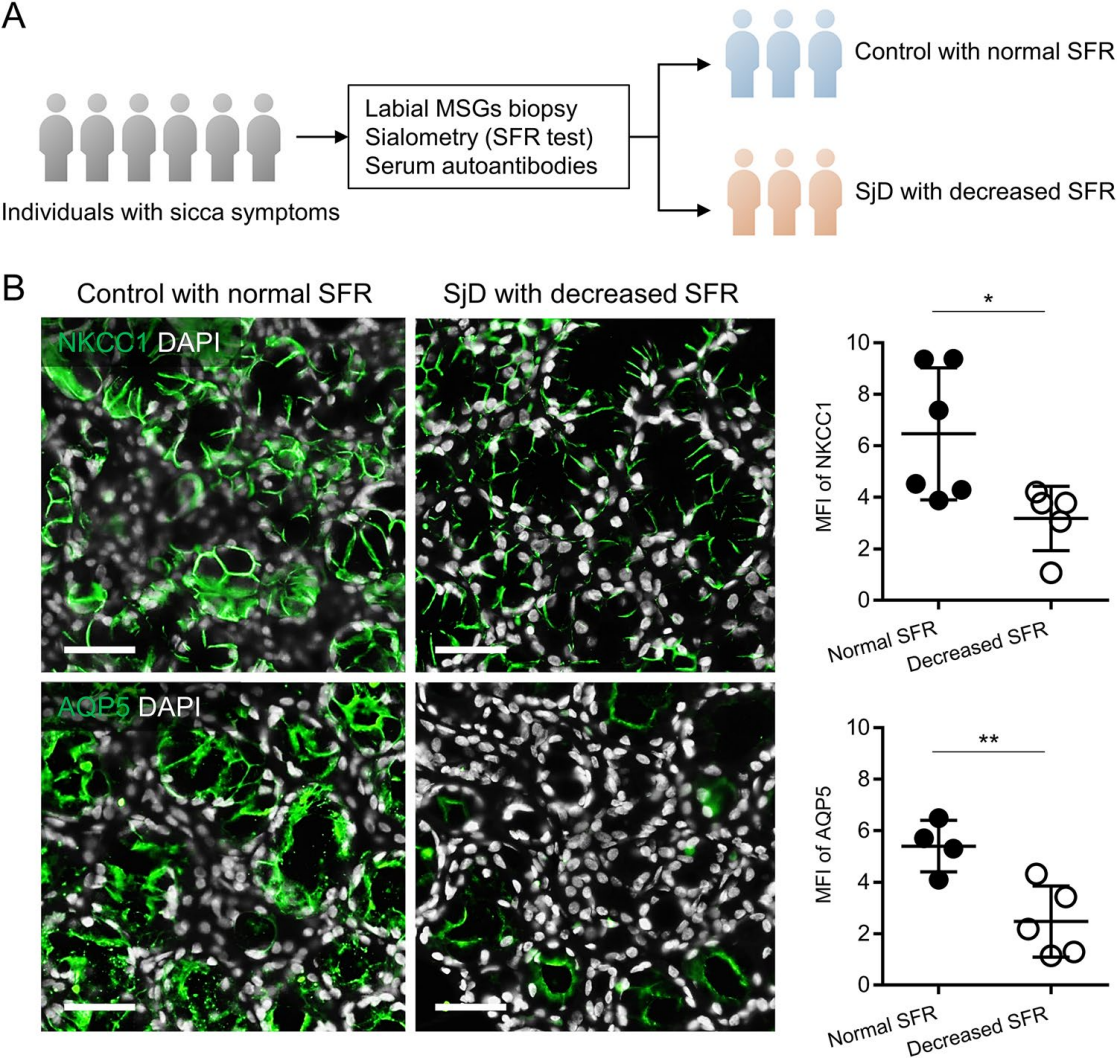
Decreased NKCC1 and AQP5 expressions are associated with salivary gland hypofunction in SjD patients. (**A**) Individuals who visited a rheumatology clinic because of sicca symptoms underwent labial minor salivary glands (MSGs) biopsy, measurement of salivary flow rate (SFR) and serum anti-Ro/SSA antibody tests. Thereafter, participants were divided into two groups: SjD patients with decreased SFR (*n* = 5) and control subjects with normal SFR (*n* = 6). (**B**) Representative immunofluorescent images of MSGs indicating staining for NKCC1 or AQP5 (green) and dot plots showing mean fluorescent intensity (MFI) of NKCC1 or AQP5 staining in each MSG (scale bars = 50 µm). Values shown are mean ± SD. *P < 0.05, **P < 0.01 (Student’s *t*-tests).

### LAMP3 promotes degradation of NKCC1 and AQP5 via endolysosomal pathway in vitro

Cell membrane proteins such as NKCC1 and AQP5 are generally degraded through the endolysosomal pathway^13^. Our previous analysis of the transcriptome of salivary glands from LAMP3-overexpressing mice showed no significant change in *Slc12a2* (encoding NKCC1) and *Aqp5* mRNA expressions, despite lower protein levels of NKCC1 and AQP5, and an increase in endosome- and lysosome-associated gene expressions^6^. This transcriptome analysis generates a hypothesis that LAMP3 can promote degradation of NKCC1 and AQP5 at the post-transcriptional level through activation of the endolysosomal pathway. In the current study, we further tested the hypothesis in vitro.

LAMP3 overexpression in Human Salivary Gland (HSG) cells decreased NKCC1 and AQP5 protein levels, as assessed by Western blotting analysis (Fig. 2A), despite increased *SLC12A2* and *AQP5* mRNA levels (Fig. 2B). Furthermore, we found significantly decreased NKCC1 and AQP5 expressions in LAMP3-overexpressing HSG cells following treatment with cyclohexamide—a protein synthesis inhibitor—compared to empty plasmid-transfected cells (Fig. 2C). Increased degradation of NKCC1 and AQP5 by LAMP3 overexpression in vitro is consistent with our previous findings in LAMP3-overexpressing mice^6^.

**Figure 2:**
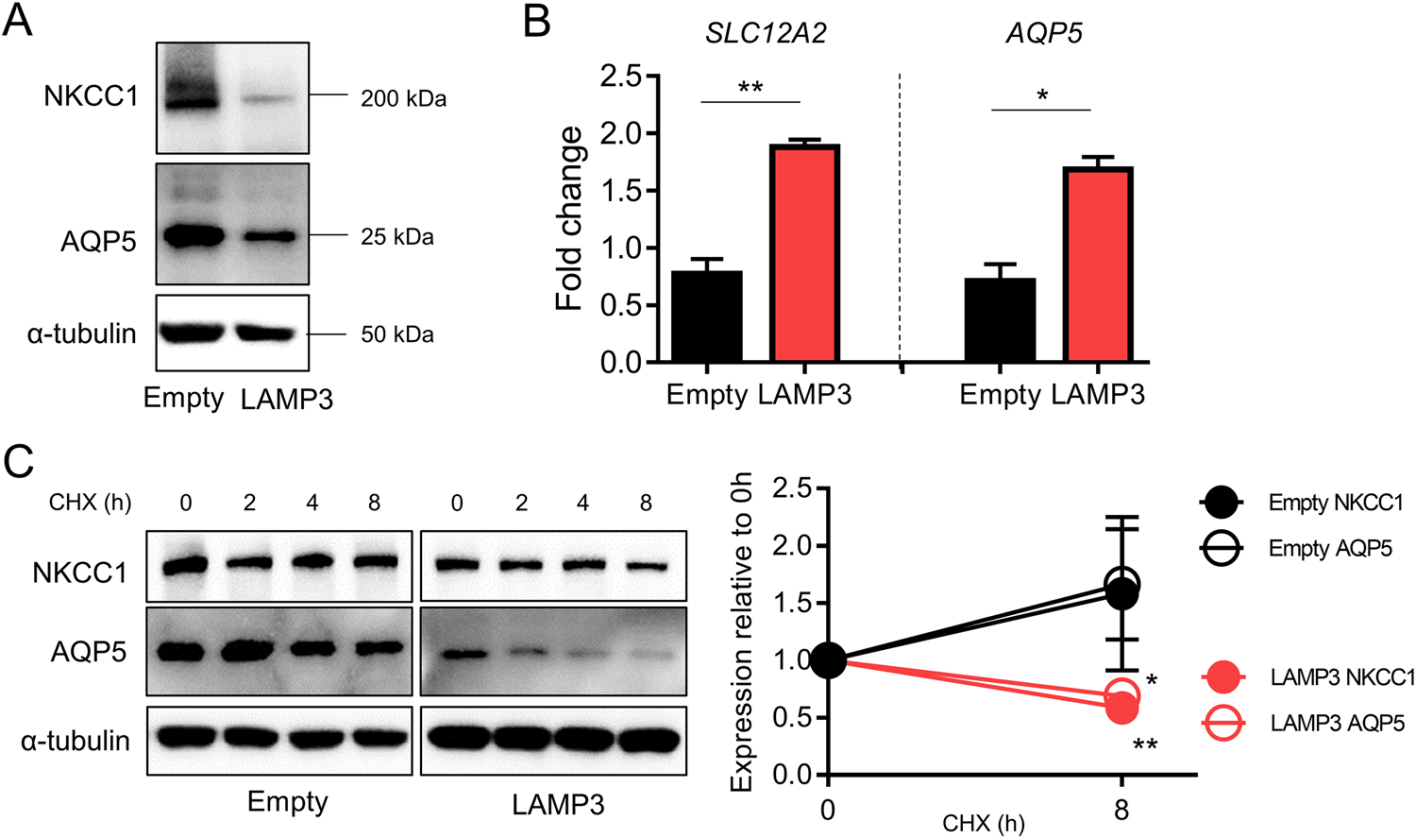
LAMP3 promotes degradation of NKCC1 and AQP5 in vitro. HSG cells were transfected with LAMP3 expression or empty plasmid (3.0 μg per 1 × 10^6^ cells). (**A**) Representative cropped Western blots showing expression of NKCC1, AQP5, or α-tubulin (internal control) 48 h after transfection. (**B**) Relative change in *SLC12A2* and *AQP5* mRNA expressions to *ACTB* expression determined by real-time quantitative PCR 48 h after transfection. (**C**) Representative cropped Western blots after cells were treated with 50 μg/mL cycloheximide (CHX) for indicated hours. Graph shows remaining protein expression after 8 h relative to baseline. Values shown are mean ± SEM (n = 3 for all experiments). *P < 0.05, **P < 0.01 (Student’s *t*-tests). Original uncropped blots are presented in Supplementary Fig. 1.

To examine whether LAMP3 overexpression stimulates the endolysosomal pathway, LAMP3-overexpressing HSG cells were incubated with Alexa Fluor 647-conjugated BSA and DQ-BSA (fluorogenic substrate for proteases), and the signals were quantified by flow cytometry and observed using a fluorescence microscope. LAMP3 overexpression significantly increased both the intensity of Alexa Fluor 647 signal uptake into the cells via endocytosis and the dequenching of the DQ-BSA signal upon endolysosomal degradation (Fig. 3). These results showed that LAMP3 overexpression promotes endocytosis and endolysosomal degradation.

**Figure 3:**
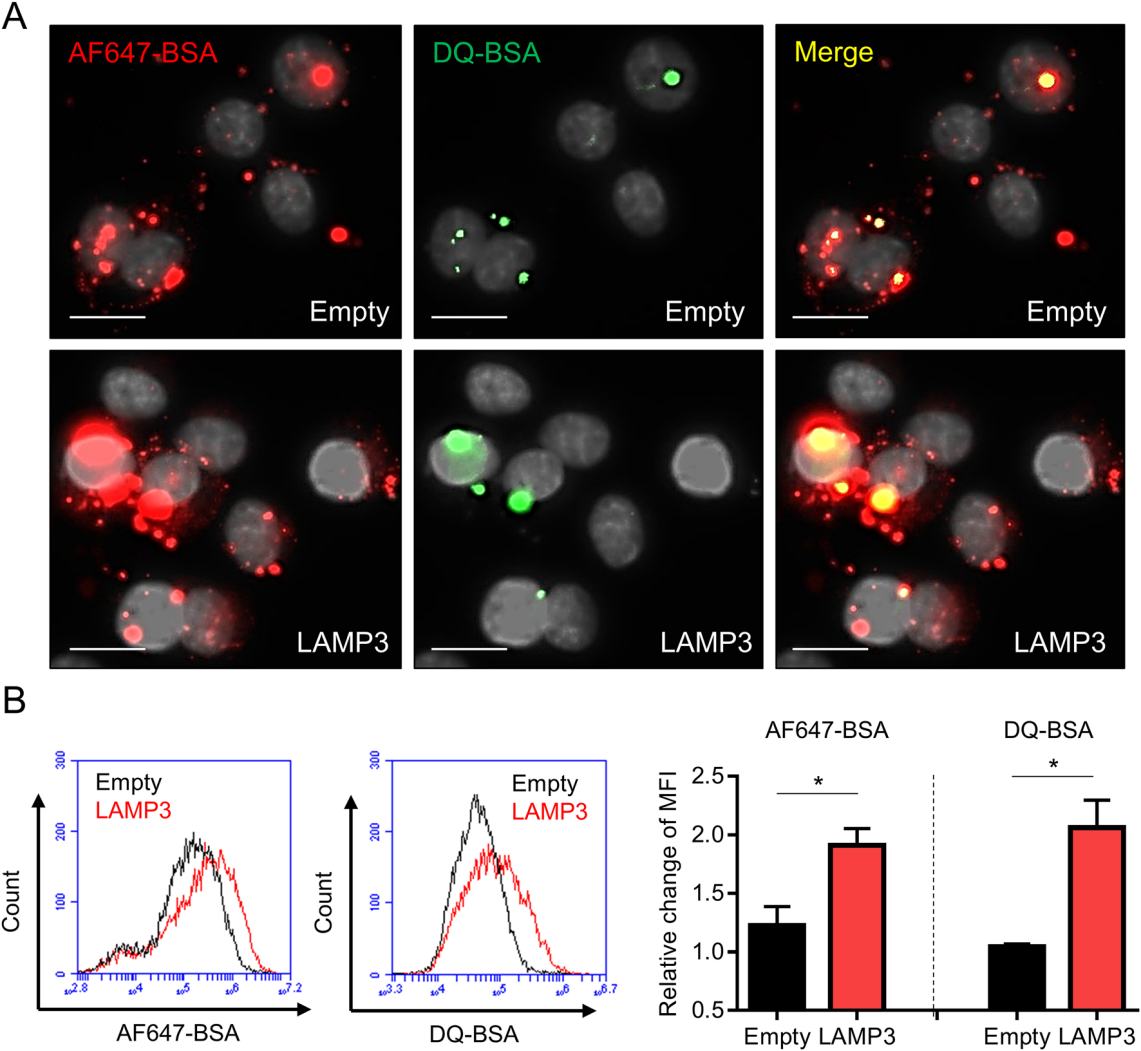
LAMP3 activates endolysosomal pathway in vitro. HSG cells were transfected with LAMP3 expression or empty plasmid (3.0 μg per 1 × 10^6^ cells) and 24 h thereafter incubated with Alexa Fluor 647 (AF647)-conjugated BSA and DQ-BSA for 4 h. (**A**) Representative immunofluorescent images showing AF647-BSA (red), DQ-BSA (green), and yielded yellow signal (scale bars = 20 µm). (**B**) Intracellular fluorescent signals were quantified by flow cytometry. Bar charts show relative change in mean fluorescent intensity (MFI). Values shown are mean ± SEM (n = 3 for all experiments). *P < 0.05 (Student’s *t*-tests).

To further investigate the LAMP3-induced increase of endolysosomal degradation of membrane proteins, LAMP3-overexpressing HSG cells were co-stained for early endosome antigen 1 (EEA1), NKCC1, and LAMP3. Immunocytochemical analysis showed that LAMP3 overexpression stimulates accumulation of EEA1 protein (Fig. 4A,B), and co-localization of EEA1 and NKCC1 (Fig. 4A,C), suggesting that LAMP3 promotes endocytosis of NKCC1 protein. Treatment with chloroquine (CQ) —an inhibitor of the endolysosomal pathway—decreased EEA1 accumulation (Fig. 4A,B) and EEA1/NKCC1 co-localization (Fig. 4A,C), resulting in an increase in NKCC1 expression (Fig. 4A,D). Western blotting analysis showed that LAMP3 overexpression decreased NKCC1 and AQP5 protein expressions in these cells, which was reversed by CQ treatment (Fig. 4E). These findings demonstrated that LAMP3 overexpression promotes NKCC1 and AQP5 degradation by activating the endolysosomal pathway.

**Figure 4:**
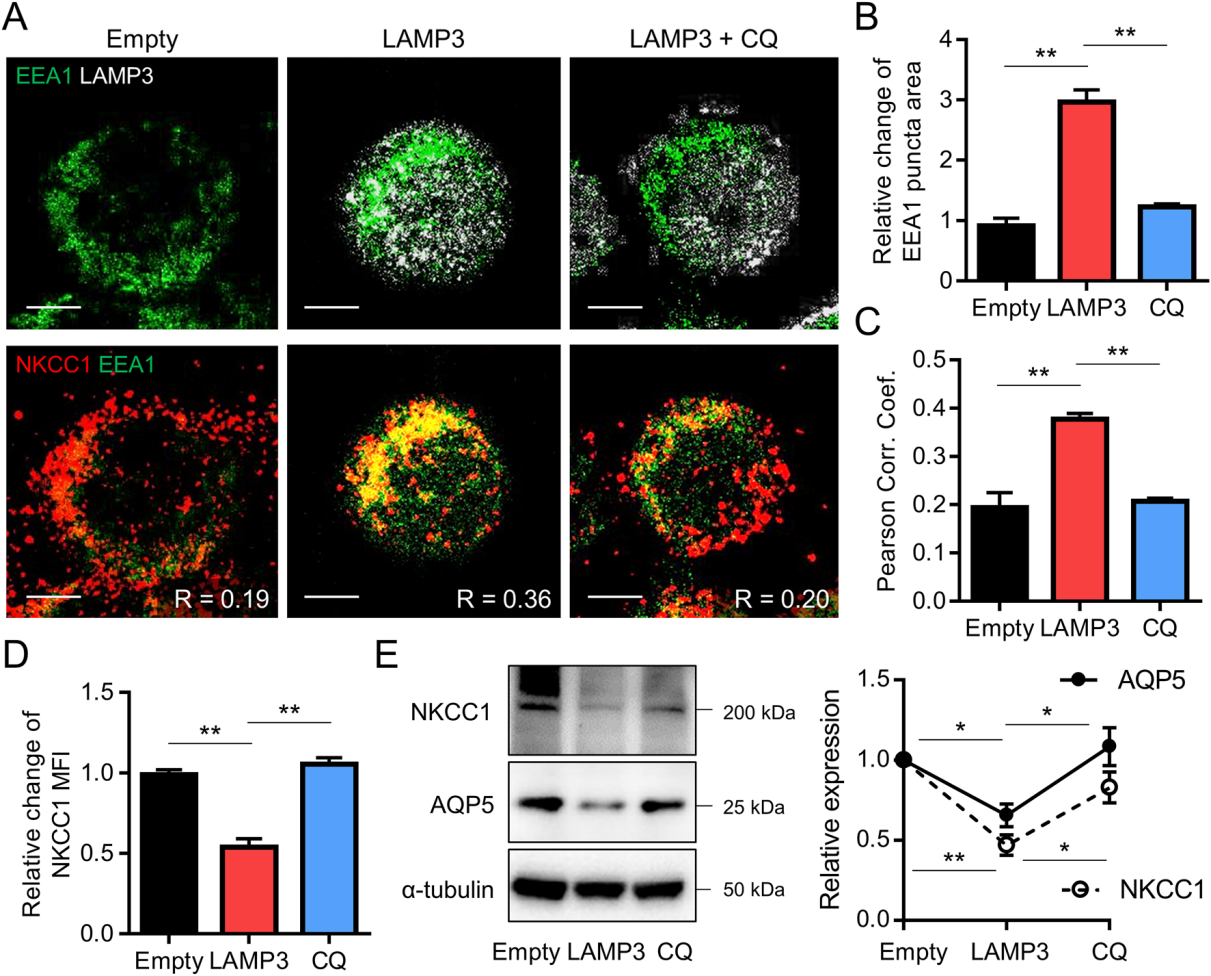
LAMP3 increases degradation of NKCC1 and AQP5 by promoting endolysosomal degradation in vitro. HSG cells were transfected with LAMP3 expression or empty plasmid (3.0 μg per 1 × 10^6^ cells) and 48 h thereafter treated with chloroquine (CQ) at 25 μM for 6 h. (**A**) Representative immunofluorescent images for LAMP3 (gray) or NKCC1 (red) and EEA1 (green) (scale bars = 5 µm). (**B**–**D**) Bar charts showing (**B**) relative change in EEA1 puncta area size, (**C**) Pearson correlation coefficients between EEA1 and NKCC1, and (**D**) relative change in mean fluorescent intensity (MFI) of NKCC1 staining (*n* = 3 for all experiments). (**E**) Representative cropped Western blots showing expression of NKCC1, AQP5, and α-tubulin (internal control). Graph shows NKCC1 and AQP5 expression relative to empty (*n* = 3). Values shown are mean ± SEM. **P < 0.01 Student’s *t*-tests; Bonferroni correction used to correct for multiple testing). Original uncropped blots are presented in Supplementary Fig. 1.

### LAMP3 promotes endolysosomal degradation of NKCC1 and AQP5 in vivo

To examine whether LAMP3 decreases NKCC1 and AQP5 expressions via the endolysosomal pathway in vivo as well as in vitro, we treated LAMP3-overexpressing mice with weekly intraperitoneal injections of hydroxychloroquine (HCQ), a derivative of CQ. Immunofluorescent analysis of salivary gland tissue showed that HCQ treatment significantly increased NKCC1 and AQP5 expressions in salivary glands of LAMP3-overexpressing mice (Fig. 5A–C). HCQ treatment also induced a significant increase in pilocarpine-stimulated SFR in LAMP3-overexpressing mice (Fig. 5D), consistent with the recovered NKCC1 and AQP5 expressions. Taken together, the in vivo results suggested LAMP3 overexpression causes salivary gland hypofunction by promoting endolysosomal degradation of NKCC1 and AQP5.

**Figure 5:**
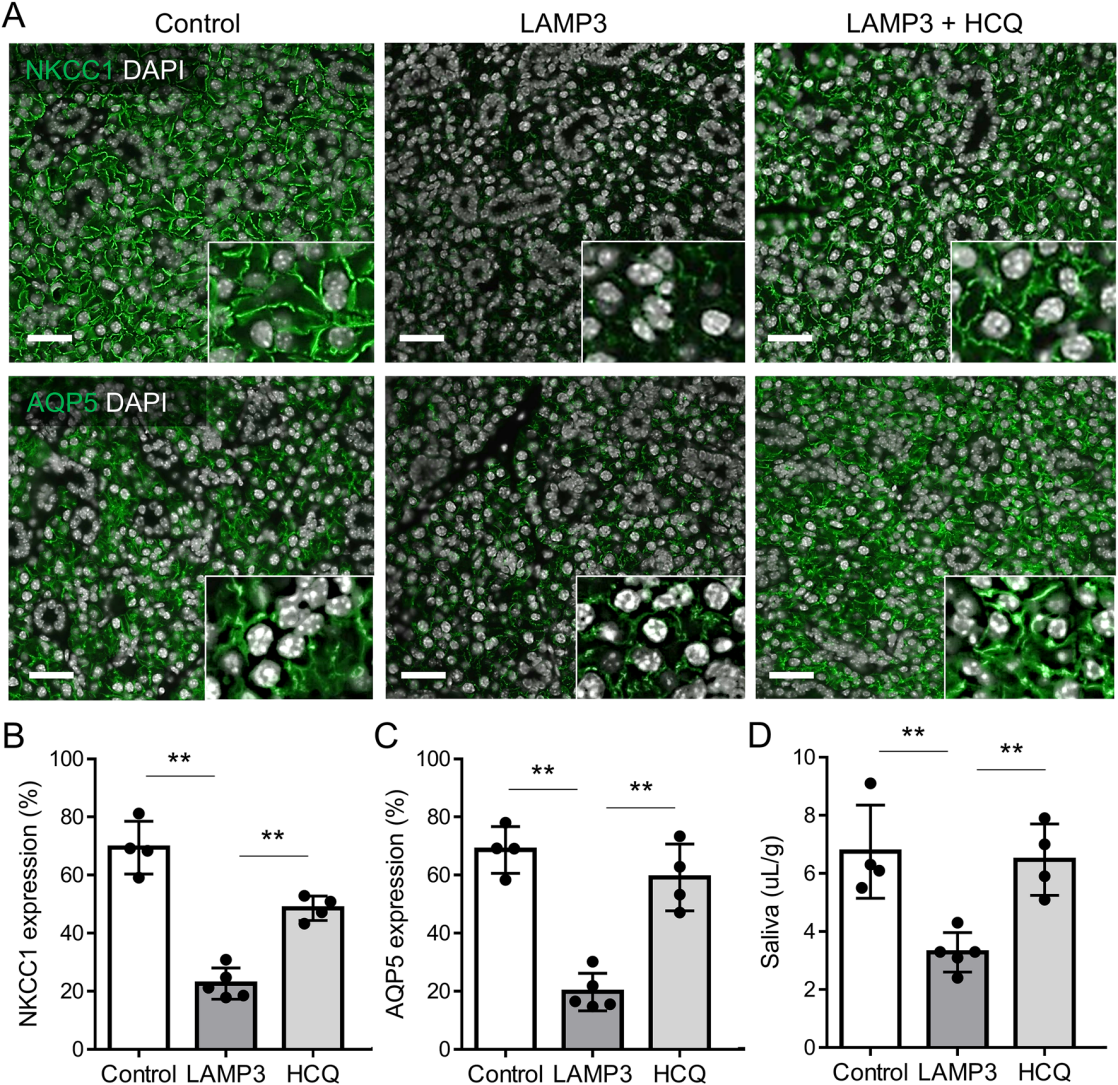
LAMP3 causes salivary gland hypofunction through endolysosomal degradation of NKCC1 and AQP5 in vivo. Submandibular glands of C57BL/6 mice were instilled with AAV2-LAMP3 or AAV2-GFP (control), after which mice received weekly intraperitoneal injections of hydroxychloroquine (HCQ) at 60 mg/kg for 4 months. (**A**) Representative immunofluorescent images of submandibular glands showing staining for NKCC1 or AQP5 (both green) (scale bars = 20 µm). (**B**, **C**) Bar charts showing percentage of (**B**) NKCC1 and (**C**) AQP5 expressions per nuclear area in submandibular gland specimens. (**D**) Pilocarpine-stimulated salivary flow rate per body weight in 20 min. Values shown are mean ± SD (4 control, 5 LAMP3-overexpressing, and 4 HCQ-treated LAMP3-overexpressing mice). **P < 0.01 Student’s *t*-tests; Bonferroni correction used to correct for multiple testing).

### LAMP3 increases AAV2-induced gene transduction by activated endocytosis

Considering the pathogenetic mechanism of salivary gland hypofunction caused by LAMP3 overexpression, replacing decreased NKCC1 and AQP5 expressions is a promising approach for restoring gland function in SjD patients. Gene therapy is a technique that transfers genetic material into cells to replace the lack of functional proteins^14^. AAV, a small, non-enveloped virus with a linear single-stranded DNA genome, is one of the most actively investigated gene therapy vehicles because of its usability and ability to transfer genes into a variety of cell types^15^. AAV transduction is dependent on proper intracellular trafficking via the endolysosomal pathway to reach the nucleus and express the encoded gene^16^. As we observed LAMP3 activates the endolysosomal protein degradation, we investigated the effect of LAMP3 on AAV transduction.

AAV transduction efficiency was compared between LAMP3-overexpressing and control HSG cells. After incubation with AAV2-GFP particles, the number of GFP-positive cells in the LAMP3-overexpressing cell culture was significantly higher than in the control cell culture (Fig. 6A), which suggested that LAMP3 enhances AAV transduction.

**Figure 6:**
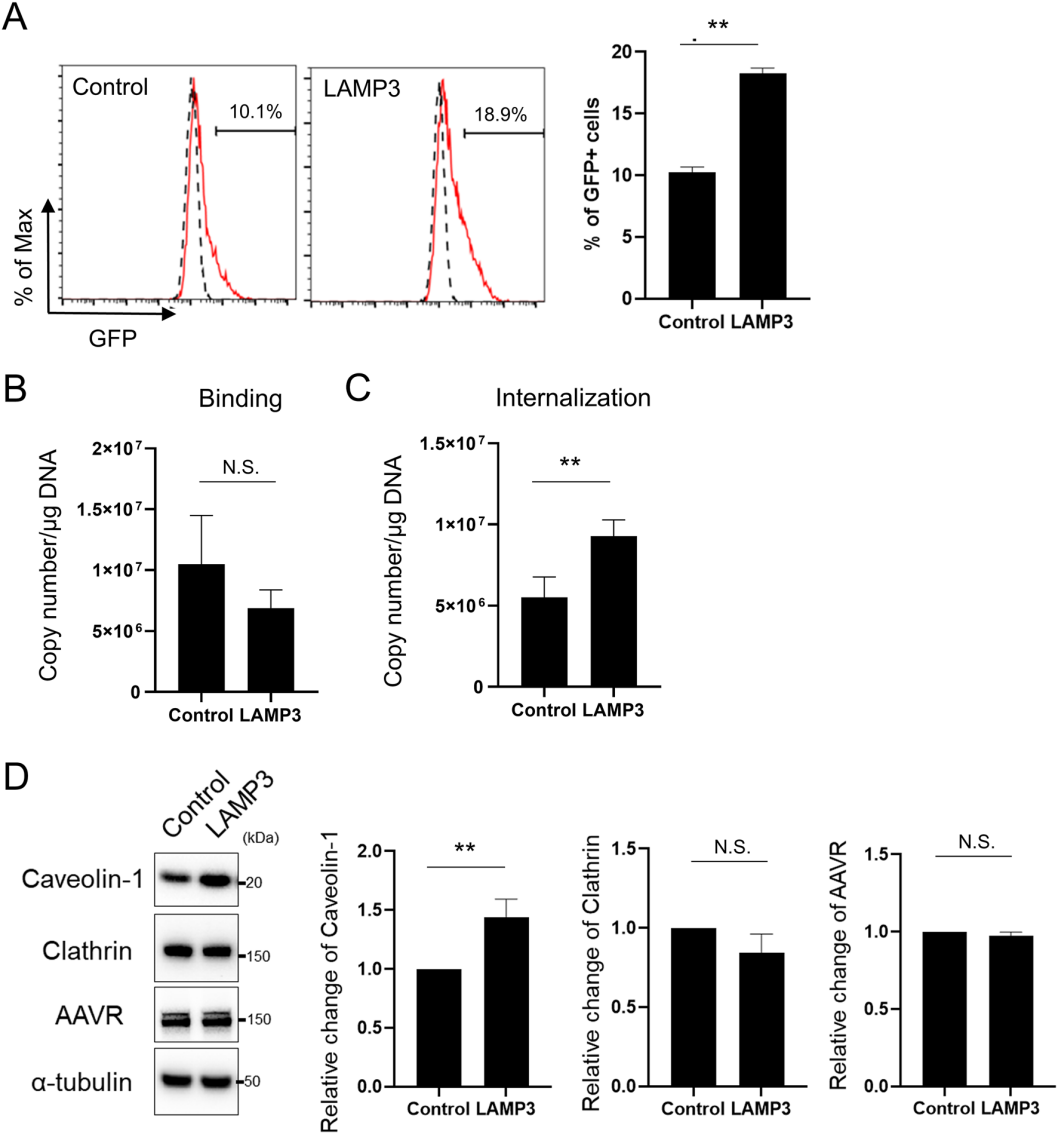
LAMP3 increases internalization of adeno-associated virus and its gene transduction in vitro. (**A**–**C**) Stably LAMP3-overexpressing and control HSG cells were incubated with AAV2-GFP particles (multiplicity of infection: 50,000) for 1 h. (**A**) Quantification of intracellular GFP signaling by flow cytometry after 48 h. Number of viral copies (**B**) that are bound on the cell membrane and (**C**) that are internalized into cells per 1 μg of DNA. (**D**) Representative cropped Western blots showing expression of caveolin-1, clathrin, KIAA0319L (AAVR), and α-tubulin (internal control) in stably LAMP3-overexpressing and control HSG cells. Bar charts showing relative expression of indicated proteins. Values shown are mean ± SEM (*n* = 3 for all experiments). **P < 0.01, N.S. = not significant (Student’s *t*-tests). Original uncropped blots are presented in Supplementary Fig. 1.

To better understand the mechanism of AAV transduction associated with LAMP3, we studied the initial steps in the AAV transduction process. Although LAMP3 overexpression did not affect binding of AAV2-GFP particles to the cell plasma membrane (Fig. 6B), it did significantly increase internalization of the viral vector (Fig. 6C). These results suggested that LAMP3 can assist AAV transduction by enhancing internalization.

To clarify the molecular details of the LAMP3-enhanced internalization of AAV, we evaluated endocytosis-related protein expression in LAMP3-overexpressing cells. Western blotting analysis showed that LAMP3 overexpression significantly increased expression of caveolin-1, but not of clathrin (Fig. 6D). Consistent with the results from the binding assay, LAMP3 overexpression did not alter expression of dyslexia-associated protein KIAA0319-like protein (KIAA0319L, also named AAV receptor), a protein associated with AAV2 binding (Fig. 6D).

### AAV2-AQP1 gene therapy restores salivary flow rate in LAMP3-overexpressing mice

The above data suggests that gene therapy via AAV vectors can be a possible way to restore gland function. Given that LAMP3 was likely inducing salivary gland hypofunction by downregulating multiple proteins involved in salivation, it seemed the best approach to create a new pathway for fluid movement to be initiated in the salivary glands. Recent clinical and preclinical work has suggested that *AQP1* gene therapy can create a new pathway for fluid movement in patients with radiation-induced salivary gland hypofunction^17^.

To test whether AAV-mediated gene therapy is effective for treating LAMP3-associated salivary gland hypofunction, we investigated the effect of gene therapy with AAV2-AQP1 in LAMP3-overexpressing mice, which develop an SjD-like phenotype with progressive salivary gland hypofunction, anti-Ro/SSA antibody production, and lymphocytic infiltration^6^. AAV2-AQP1 or control AAV2-GFP was also delivered to the submandibular glands of C57BL/6 mice by retrograde cannulation 7 months after retrograde ductal instillation of AAV2-LAMP3 (Fig. 7A).

**Figure 7:**
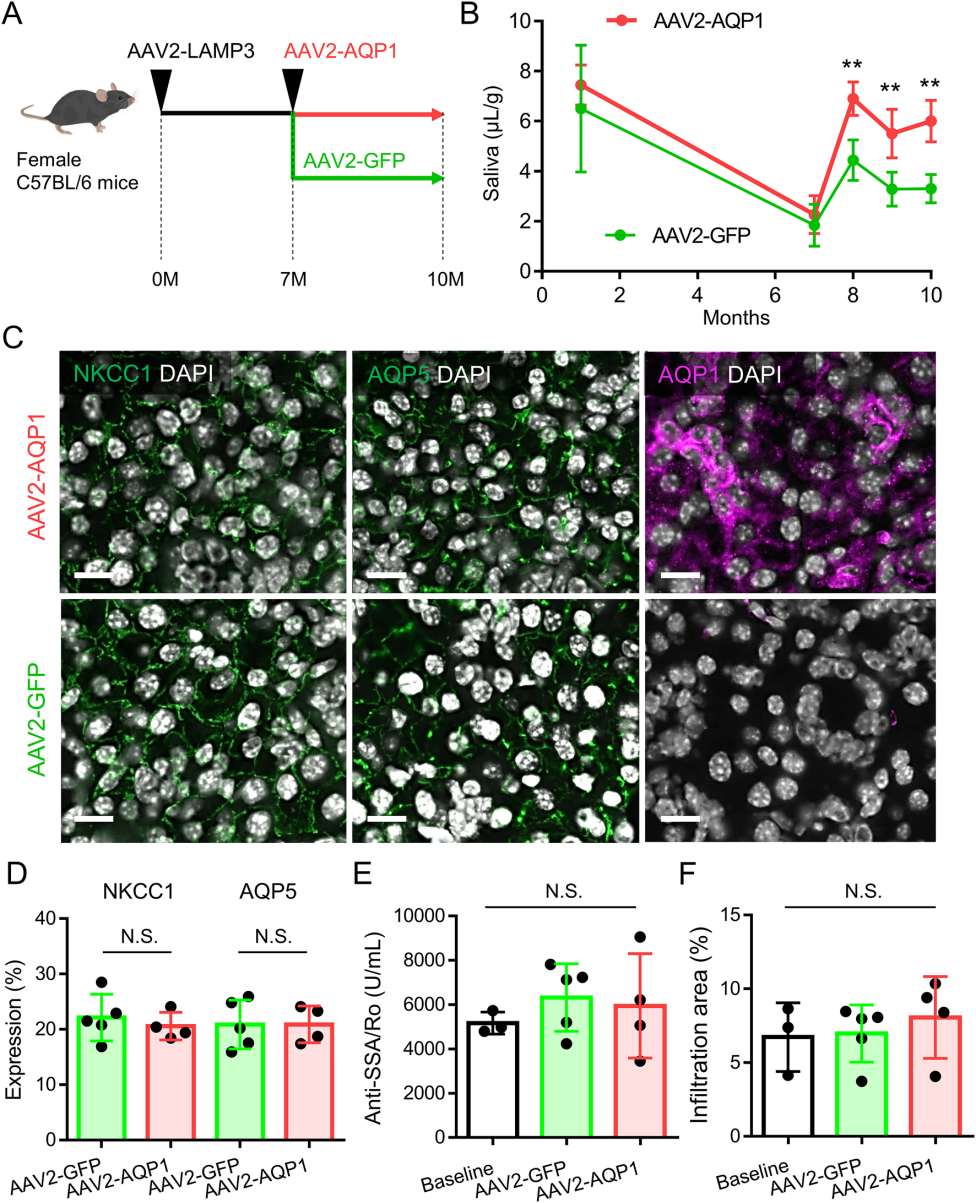
AAV2-AQP1 gene therapy restores salivary flow rate in LAMP3-overexpressing mice. (**A**) LAMP3 overexpression in submandibular glands of C57BL/6 mice was achieved by retrograde cannulation of AAV2-LAMP3. After 7 months, AAV2-AQP1 or AAV2-GFP (control) was delivered to submandibular glands of LAMP3-overexpressing mice by retrograde cannulation. (**B**) Pilocarpine-stimulated salivary flow rate per body weight in 20 min in mice. (**C**) Representative immunofluorescent images of submandibular glands for NKCC1 (green), AQP5 (green), or AQP1 (magenta) (scale bars = 10 µm). (**D**) NKCC1 and AQP5 expression area in glands. (**E**) Serum anti-SSA/Ro antibody levels. (**F**) Size of lymphocyte infiltration area in glands. Values shown are mean ± SD (3 baseline, 5 AAV2-GFP-treated, and 4 AAV2-AQP1-treated mice). **P < 0.01, N.S. = not significant, Student’s *t*-tests; Bonferroni correction used to correct for multiple testing).

Treatment with AAV2-AQP1 significantly restored pilocarpine-stimulated SFR compared to control treatment (AAV2-GFP), which was visible after 1 month and lasted for at least 3 months (Fig. 7B). While AAV2-AQP1 treatment induced glandular AQP1 protein expression in AAV2-AQP1-treated mice as compared to control mice, it did not affect NKCC1 and AQP5 expressions (Fig. 7C,D). AQP1 expression was predominantly localized in salivary gland epithelial cells (Fig. 7C), consistent with the tropism of AAV2^18,19^. Treatment with AAV2-AQP1 did not significantly change serum anti-Ro/SSA antibody levels nor the size of the lymphocytic infiltration areas in the glands compared to control treatment (AAV2-GFP) and baseline (Fig. 7E,F). These results are graphically summarized in Fig. 8.

**Figure 8:**
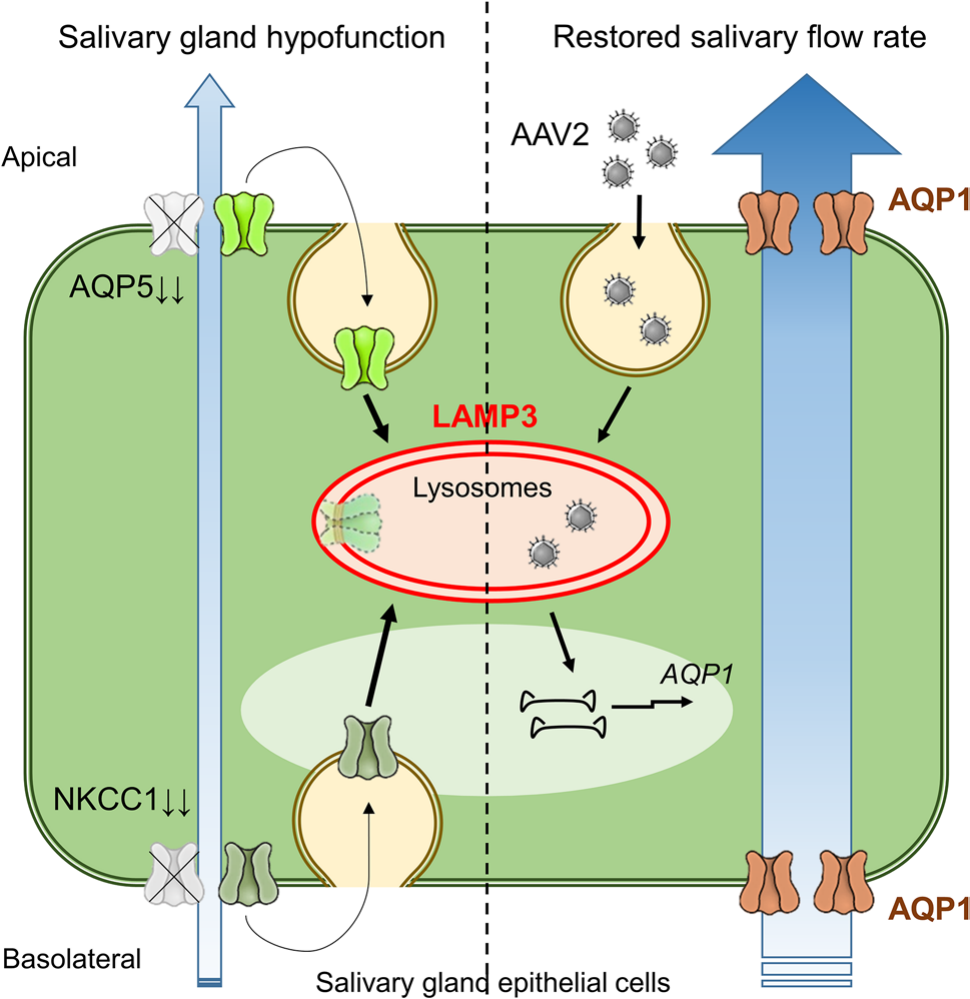
Graphical summary. LAMP3 overexpression decreases AQP5—a water channel in the apical membrane—and NKCC1—an ion cotransporter on the basolateral membrane—expressions in salivary gland epithelial cells by promoting endolysosomal degradation, resulting in salivary gland hypofunction. Simultaneously, LAMP3 increases internalization of AAV2 and enhances AAV2-mediated gene transduction efficiency via the activated endolysosomal pathway. AAV2-mediated *AQP1* gene therapy induces ectopic AQP1 expression in both apical and basolateral membranes of salivary gland epithelial cells and creates a new pathway for fluid movement, leading to restoration of salivary flow rate in LAMP3-overexpressing mice.

## Discussion

In the present study, we found that LAMP3 overexpression activates the endolysosomal pathway in salivary gland epithelial cells as a novel mechanism of action. In addition, we demonstrated that LAMP3 plays an important pathophysiological role in the development of salivary gland hypofunction associated with SjD by promoting endolysosomal degradation of NKCC1 and AQP5. These findings lead to a hypothesis that we would need to introduce a new mechanism for initiating fluid movement in the lumen of the glands in order to restore LAMP3-associated salivary gland hypofunction. In this study, we have tested the hypothesis by introducing the water channel AQP1 into the salivary gland epithelial cells. Interestingly, LAMP3 overexpression increased AAV infection and its gene transduction efficiency via the promoted endolysosomal pathway. Our findings suggest it is possible to increase fluid movement in this model following AAV-mediated *AQP1* gene transfer.

Although SjD is considered to be a systemic disease, patients’ main complaints are the severe sicca symptoms (indicating loss of secretory function) and the impact these have on many aspects of daily life^2^. To meet patients’ needs, therapies targeting specific localized features have been investigated. Gene delivery of molecules, such as soluble CTLA4IgG fusion protein and modulator of the interleukin-17 pathway, to salivary glands of SjD mouse models have shown promise^20–22^. An advantage of gene therapy to the salivary glands is that delivery of local immunomodulators via gene transfer results in a higher local concentration and a comparable treatment effect while minimizing the side effects associated with systemic delivery of the same drug molecule.

Over the past 10 years, the field of gene therapy has advanced from concept to clinical stage and drug approval. For example, in a phase 1 clinical trial, adenovirus-mediated *AQP1* gene transfer successfully restored salivary gland function in patients with chronic xerostomia who had undergone ionizing radiation therapy for treatment of head and neck cancer^17^. In this trial, an adenoviral vector was injected into the parotid gland, but it is not clear if this is the best vector for gene transfer to the salivary glands. Another clinical trial using AAV2-mediated *AQP1* transfer to the salivary glands of the same patient population is currently ongoing (NCT02446249). Its results are expected to offer more information on dosing AAV2 vector and may support application of this treatment in SjD.

AQPs are a new drug target because their dysfunction is implicated in numerous pathologies, such as tumors, ischemic diseases, and traumatic injury, in addition to SjD^23^. For instance, AQP4 expression in the blood–brain and blood-spinal cord barriers is associated with brain or spinal cord edema after stroke or spinal cord injury^24,25^. However, no AQP-targeted drugs have been approved for clinical use yet^26^. The current study suggested AAV2-mediated gene transfer as an alternative way to target AQP function. Previous research has demonstrated that AAV transduction can be blocked by disease processes that affect critical steps in the transduction pathway^15^. But, LAMP3 misexpression in SjD patients’ salivary glands may have an advantage for AAV2-mediated gene transfer as we showed LAMP3 enhances AAV2 transduction by activating the endolysosomal pathway.

Bone morphogenetic protein 6 (BMP6) is another protein that is upregulated in SjD patients’ salivary glands and is associated with the pathophysiology^27^. Previously, we reported that salivary gland epithelial LAMP3 expression stimulates BMP6 production by monocytes via the extracellular release of HSP70 and via activation of Toll-like receptor 4^28^. BMP6 decreases *AQP5* expression at the transcriptional level^27–29^. When combining these data with the LAMP3-mediated post-transcriptional degradation of AQP5 that we identified, we could argue that LAMP3 and BMP6 downregulate AQP5 expression together at both transcriptional and post-transcriptional levels, resulting in salivary gland hypofunction. In BMP6-overexpressing mice, AAV2-mediated expression of AQP1 was also able to restore SFR^30^.

Viral infection and the subsequent immune response, especially the induction of interferon, are closely related to the development of autoimmunity in SjD^31,32^. Therefore, gene therapy using viral vectors could pose a potential risk of worsening autoimmunity in SjD. AAV, which is a non-pathogenic virus, causes a very mild immune response in humans^33^. We observed no significant increases in serum autoantibodies against Ro/SSA or glandular lymphocytic infiltration in an SjD mouse model following treatment with AAV2-AQP1 compared to baseline.

In conclusion, we found that salivary gland hypofunction in SjD is associated with decreased NKCC1 and AQP5 expressions in salivary gland epithelial cells in which LAMP3 expression activates the endolysosomal pathway and promotes these protein degradation. Also, we discovered that the activated endolysosomal pathway by LAMP3 increases AAV transduction and demonstrated that AAV2-AQP1 gene therapy ameliorates LAMP3-associated salivary gland hypofunction in an SjD mouse model. Our findings suggest that AAV2-AQP1 gene therapy could be useful in the management of salivary gland hypofunction in SjD.

## Methods

### Patients

Study participants were recruited at the Hokkaido University Hospital in Sapporo, Japan. Clinical samples including labial minor salivary gland, saliva and serum were collected in accordance with the Declaration of Helsinki principles. All the protocols were approved by the Ethics Committee of the Hokkaido University Hospital (Approval number: 014-0466). Informed consent was obtained from all participants. Clinical profiles of the participants are summarized in Supplementary Table 1.

### Animals

LAMP3-overexpressing mice were generated by administration of AAV2 vector encoding the gene for *LAMP3* (AAV2-LAMP3) using retrograde cannulation of both submandibular glands of female 6–8 week-old C57BL/6 mice (Charles River Laboratories, USA), as described previously^6^. Control mice received AAV2 vector containing the gene for green fluorescent protein (AAV2-GFP) instead. LAMP3-overexpressing mice received a weekly intraperitoneal injection of hydroxychloroquine (HCQ, Plaquenil® tablet in a vehicle of PBS, Sanofi, USA) at 60 mg/kg body weight for 4 months, as described previously^34^. Another set of LAMP3-overexpressing mice received AAV2 vectors encoding the gene for *AQP1* (AAV2-AQP1) or AAV2-GFP (10^11^ particles/mouse in 100 μl) by retrograde cannulation of both submandibular glands, as described previously^30^. Treatment groups were blindly allocated to each cage of mice, but no randomization was performed for the allocation. All mice were analyzed without any criterion for inclusion and exclusion. Outcomes were evaluated by a blinded researcher. Confounders were not controlled for this study.

Pilocarpine-stimulated SFR in 20 min was determined at several time points post-cannulation, as described previously^6^. At the end of the study, serum and whole submandibular glands were collected. Presence of serum anti-Ro/SSA antibodies was detected using a solid-phase ELISA (#5710, Alpha Diagnostic International), according to the manufacturer’s instructions.

All procedures involving live animals were performed in accordance with institutional guidelines and standard operating procedures following the National Institutes of Health Guide for the Care and Use of Laboratory Animals. All the protocols were approved by the Ethics Committee of the National Institute of Dental and Craniofacial Research Veterinary Resources Core (approval number: 18–863). All methods are reported in accordance with ARRIVE guidelines (https://arriveguidelines.org) for the reporting of animal experiments.

### Immunofluorescent staining

Formalin-fixed, paraffin-embedded salivary gland sections were mounted on glass slides, deparaffinized, rehydrated, and subjected to citric acid microwave antigen retrieval. Slides were blocked with 2% BSA (Sigma-Aldrich) in PBS at room temperature for 30 min and incubated at 4 °C overnight with one of the following primary antibodies: 10 μg/mL of rabbit anti-NKCC1 (#4828 for human and #85,403 for mouse, Cell Signaling Technology, USA), rabbit anti-AQP5 (#AQP-005, Alomone labs, Israel), or rabbit anti-AQP1 (#ab168387, Abcam, USA).

Slides were then incubated with 10 μg/mL of Alexa Fluor 488 or Alexa Fluor 594 AffiniPure Donkey Anti-Rabbit IgG (H + L) (both from Jackson Immuno Research) at room temperature for 1 h, followed by counterstaining with DAPI mounting medium (Abcam).

For data acquisition and analysis, three to five images per sample were acquired with a fluorescent microscope (Nikon, Japan). Protein expression levels were determined by subtraction of the signals from control rabbit IgG staining (Jackson ImmunoResearch) and normalized by DAPI intensity using ImageJ software (in public domain source: National Institutes of Health, USA).

### Cells

HSG cells, which were kindly provided by Dr. Indu Ambudkar (National Institute of Dental and Craniofacial Research), were cultured in DMEM (Thermo Fisher Scientific, USA) supplemented with 10% FBS and incubated at 37 °C and 5% CO_2_. Transient transfection of LAMP3-encoding plasmid and establishment of stably LAMP3-overexpressing HSG cells were done, as described previously^5^.

HSG cells within 30 passages were used at 70–80% confluence (doubling time: 24 h) for all the experiments. Mycoplasma contamination was checked using MycoAlert Mycoplasma Detection Kit (Lonza, Switzerland) prior to the experiments.

### Immunocytochemistry

HSG cells were fixed with 4% paraformaldehyde for 15 min, permeabilized using 0.1% Triton-X-100 (Sigma-Aldrich) for 10 min, and then blocked with 2% BSA for 30 min, all at room temperature. Cells were incubated at 4 °C overnight with 10 μg/ml of primary antibodies: anti-LAMP3 antibody (#12,632-1-AP, Proteintech) directly labeled with Alexa Fluor 488, anti-EEA1 antibody (#PA1-063A, Thermo Fisher Scientific) directly labeled with Alexa Fluor 594 and anti-NKCC1 antibody (#4828, Cell Signaling Technology) directly labeled with Alexa Fluor 647. Antibodies were labeled using Lightning-Link Rapid Labeling Kit (Novus Biologicals, USA). Sections were counterstained with DAPI mounting medium (Abcam). All images were acquired using a confocal fluorescent microscope (Nikon, Japan) and analyzed using ImageJ software.

### Quantitative real-time reverse transcription PCR

Total RNA from HSG cells was extracted using the RNeasy Mini Kit (QIAGEN, USA), treated with the RNase-Free DNase Set (QIAGEN), and reverse transcribed into cDNA using the SuperScript VILO cDNA Synthesis Kit (Thermo Fisher Scientific). Real-time quantitative PCR reactions were performed using TaqMan gene expression assays (Thermo Fisher Scientific) and following TaqMan probes: *SLC12A2* (Hs00169032_m1), *AQP5* (Hs00387048_m1), and *ACTB* (Hs01060665_g1). Gene expression relative to *ACTB* was calculated using ΔΔCt method. PCR cycles were performed on the Quantstudio3 Real-Time PCR System (Life Technologies, USA) with the following conditions: 2 min at 50 ℃, 10 min at 95 ℃, 50 cycles of 15 s at 95 ℃ and 1 min at 60 ℃.

### Western blotting

HSG cells were lysed in RIPA Lysis and Extraction Buffer with additional protease and phosphatase inhibitors (all from Thermo Fisher Scientific) and cleared by centrifugation at 17,000 g at 4 °C for 25 min. Supernatants were heated at 97 °C in NuPAGE LDS Sample Buffer for 10 min, resolved by SDS-PAGE, and electrophoretically transferred to polyvinylidene difluoride membranes (Thermo Fisher Scientific). Membranes were blocked with 2% non-fat dried milk at 25 °C for 1 h and then incubated at 4 °C overnight with one of the following primary antibodies: anti-NKCC1 (#4828, Cell Signaling Technology), anti-AQP5 (#AQP-005, Alomone labs), anti-caveolin-1 (#3267, Cell Signaling Technology), anti-clathrin heavy chain (#4796, Cell Signaling Technology), anti-KIAA0319L/AAVR (#21,016-1-AP, Proteintech), or anti-α-tubulin (#T6199, Sigma-Aldrich). After washing three times, membranes were incubated with rabbit or mouse IgG horseradish peroxidase-linked whole antibody (Sigma-Aldrich) at 25 °C for 1 h. Signals were visualized using Super Signal West Pico PLUS Chemiluminescent Substrate (Thermo Fisher Scientific).

### Flow cytometry

For the endocytosis and lysosomal degradation assays, HSG cells were incubated with 10 μg/mL of Alexa Fluor 647-conjugated BSA (Thermo Fisher Scientific) and DQ-BSA (Thermo Fisher Scientific) in DMEM supplemented with 10% FBS at 37 °C and 5% CO_2_ for 4 h.

For the AAV2-GFP infection assay, HSG cells were incubated in 1 mL of medium including AAV2-GFP particles at a multiplicity of infection (MOI) of 50,000 for 1 h and then stained with 7-AAD (Biololegend, USA) after 48 h.

Signals were detected using the BD Accuri C6 Flow Cytometer (BD Biosciences, USA) and FlowJo software (BD Biosciences).

### AAV2-GFP binding and internalization assay

For the AAV2-GFP binding assay, HSG cells were pre-incubated at 4 °C for 1 h, incubated with AAV2-GFP particles (MOI: 50,000) at 4 °C for 1 h, washed once in ice-cold DMEM and twice in ice-cold PBS, and then collected by scraping.

For the AAV2-GFP internalization assay, HSG cells were incubated with AAV2-GFP particles (MOI: 50,000) in pre-warmed DMEM at 37 °C for 1 h, collected by trypsinization on ice for 15 min^35^, trypsinized at 37 °C for 5 min to eliminate virus on the membrane surface, and then washed once in ice-cold DMEM and twice in ice-cold PBS.

DNA was isolated from collected cells using the DNeasy Blood and Tissue Kit (QIAGEN), according to the manufacturer’s instructions. Copy number per 1 μg of DNA was determined using the following primers and probe designed to amplify part of the CMV promoter region: forward primer, 5’- CAT CTA CGT ATT AGT CAT CGC TAT TAC CAT -3’; reverse primer, 5’- TGG AAA TCC CCG TGA GTC A -3’; probe, 5’-/56-FAM/ACA TCA ATG GGC GTG GAT AGC GGT /36-TAMSp/ -3’ (all from Integrated DNA Technologies, Inc., USA). The conditions were as follows: 2 min at 50 °C, 10 min at 95 °C, 50 cycles of 15 s at 95 °C, and 1 min at 60 °C.

### Statistical analysis

Data are presented as mean ± standard deviation (SD) or standard error of the mean (SEM). Quantitative variables were compared using a two-tailed Student’s *t*-test and Pearson’s correlation coefficient. When appropriate, Bonferroni correction was used to correct for multiple hypothesis testing. Categorical variables were compared using the Chi-squared test. *P* values < 0.05 were considered statistically significant. All analyses were performed using GraphPad Prism 8.0 software.

### Ethical approval

Ethics Committee of the National Institute of Dental and Craniofacial Research.

## Supplementary Information

Supplementary Information.

## Data Availability

All relevant data are included in the article.
