# Supplementary material for: Correction of LAMP3-associated salivary gland hypofunction by aquaporin gene therapy
Source: Sci Rep. 2022 Nov 3;12:18570. doi: 10.1038/s41598-022-21374-2 (PMC9633788; doi:10.1038/s41598-022-21374-2)

Correction of LAMP3-associated salivary gland hypofunction by aquaporin gene therapy

Hiroyuki Nakamura[1†], Tsutomu Tanaka[1†], Changyu Zheng[1], Sandra A Afione[1], Blake M. Warner[2], Masayuki Noguchi[3], Tatsuya Atsumi[4] and John A. Chiorini[1*]

1) Adeno-Associated Virus Biology Section, National Institute of Dental and Craniofacial Research, National Institutes of Health, Bethesda, MD, USA.

2) Salivary Disorder Unit, National Institute of Dental and Craniofacial Research, National Institutes of Health, Bethesda, MD, USA.

3) Division of Cancer Biology, Institute for Genetic Medicine, Hokkaido University, Sapporo, Japan.

4) Department of Rheumatology, Endocrinology and Nephrology, Faculty of Medicine, Hokkaido University, Sapporo, Japan.

[†]These authors contributed equally to this work.

*Corresponding author: John A. Chiorini, PhD

AAV Biology Section, NIDCR, NIH 10 Center Drive, Bethesda, MD 20892, USA.

Phone: +301-496-4279. Email: jchiorini@dir.nidcr.nih.gov

Supplementary Table 1. Participant characteristics

Age (y)	Sex	Decreased SFR	Focus score ≥ 1	Anti-Ro/SSA	2016 ACR/EULAR criteria
SjD patients with decreased SFR (*n* = 5)					
64	F	Yes	Yes	No	Yes
36	F	Yes	Yes	Yes	Yes
61	F	Yes	No	Yes	Yes
35	F	Yes	Yes	Yes	Yes
23	F	Yes	Yes	Yes	Yes
Control subjects with normal SFR (*n* = 6)					
49	F	No	Yes	Yes	Yes
52	F	No	Yes	Yes	Yes
31	F	No	No	Yes	No
59	F	No	No	Yes	No
48	F	No	No	Yes	No
38	F	No	No	No	No

F, female; SFR, salivary flow rate.

Supplementary Figure 1. Original uncropped blots

Figure 2A

NKCC1

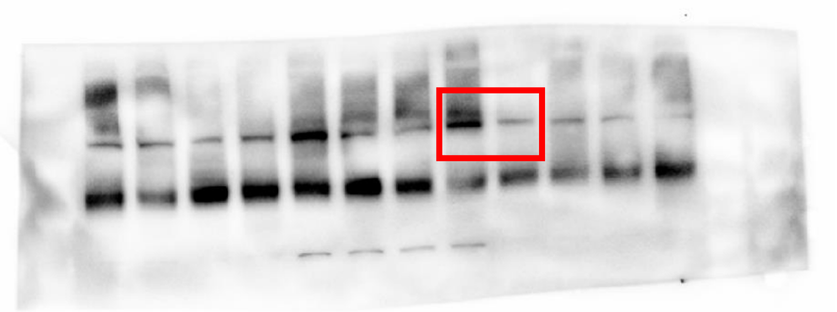

Empty LAMP3

AQP5

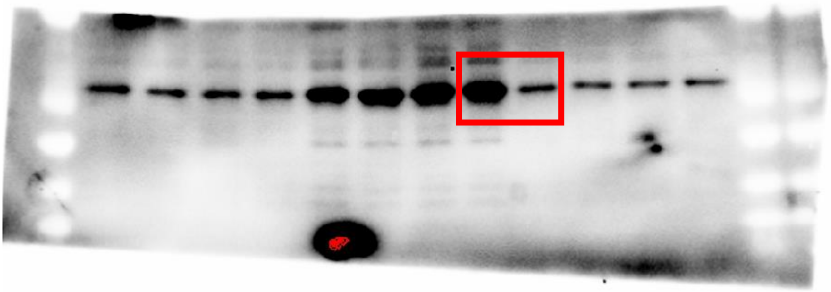

Empty LAMP3

α-tubulin

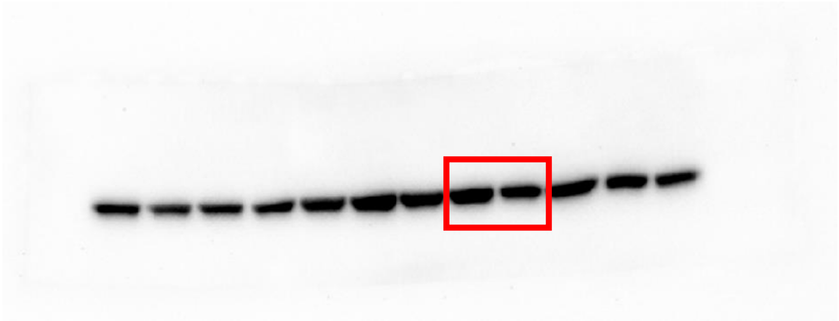

Empty LAMP3

Supplementary Figure 1. Original uncropped blots

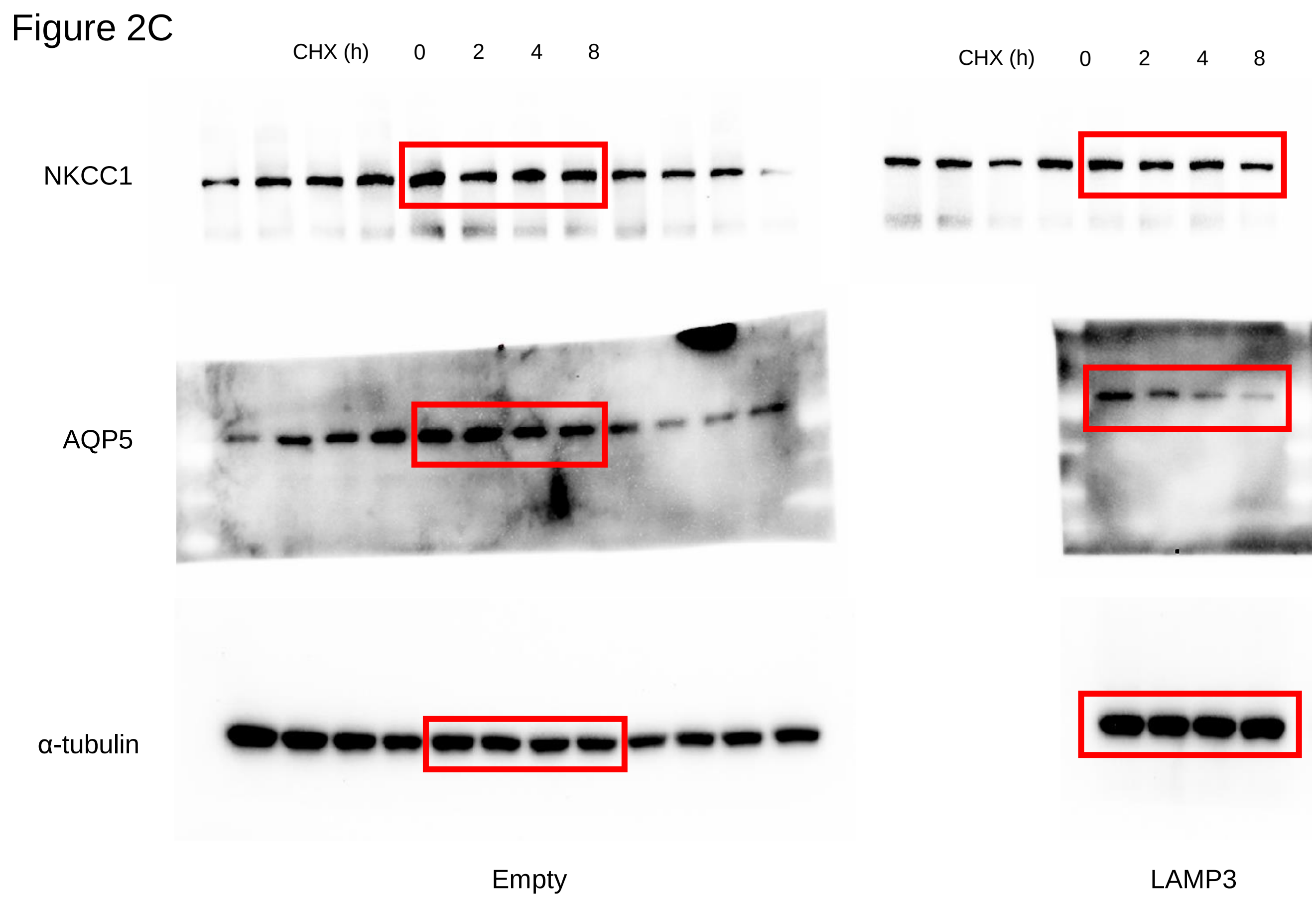

Supplementary Figure 1. Original uncropped blots

Figure 4E

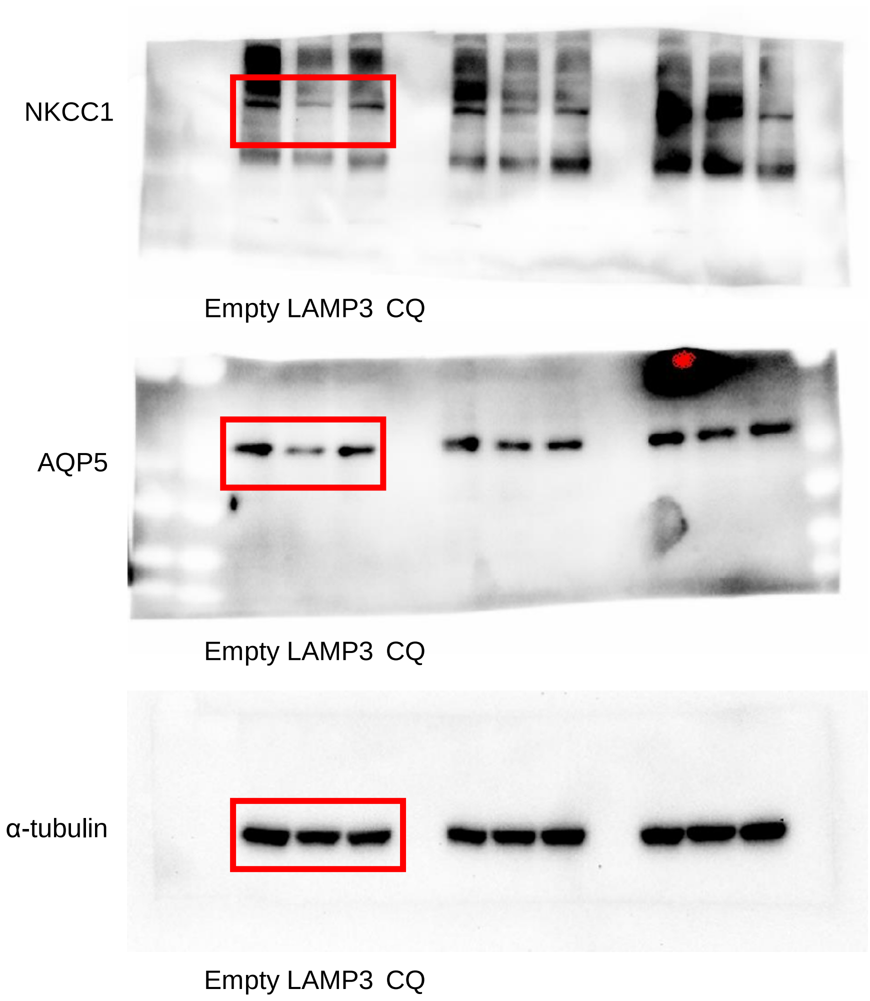

Supplementary Figure 1. Original uncropped blots

Figure 6D

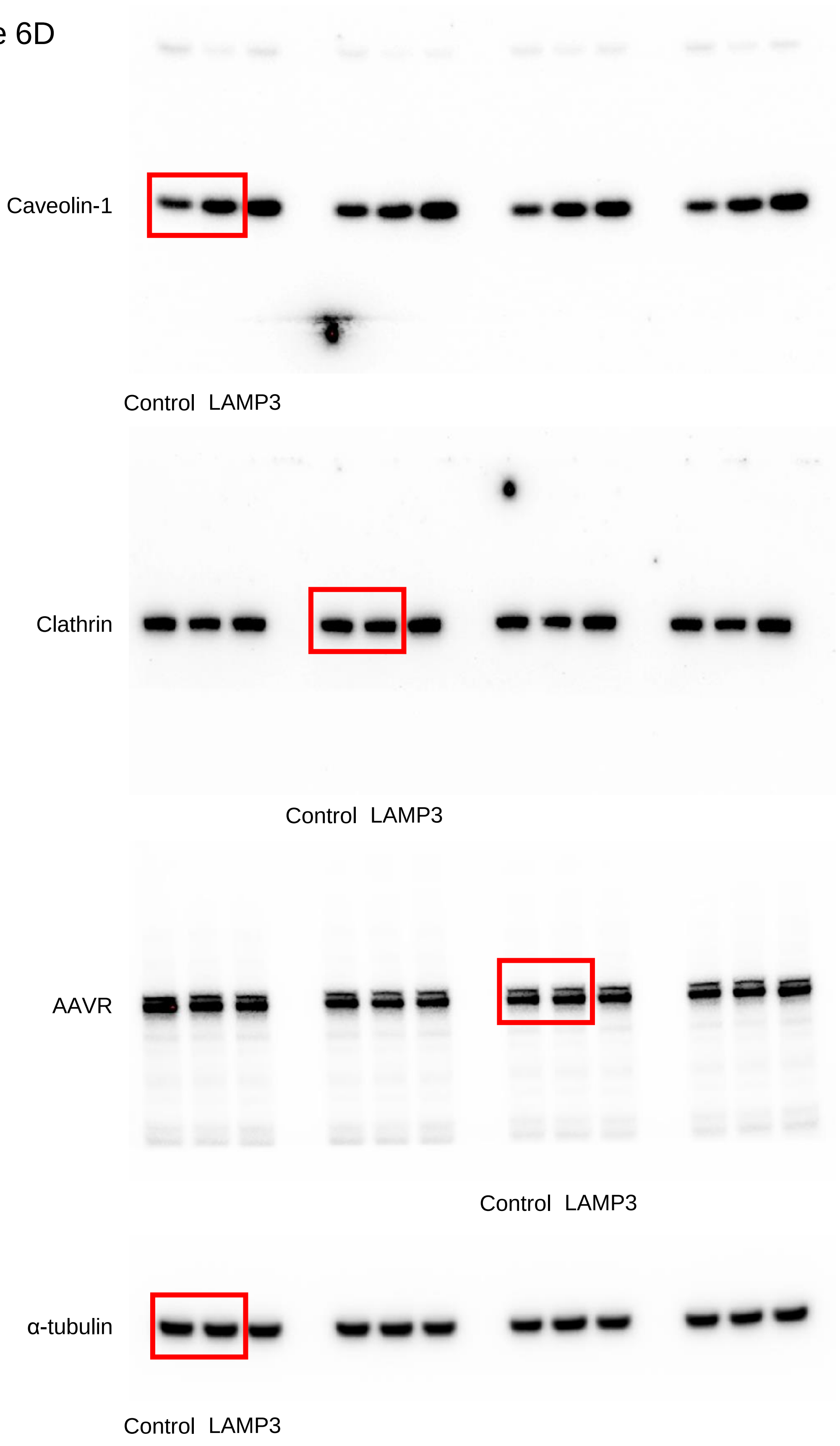

Supplement: Supplementary file 1 — Supplementary Information. [file 41598_2022_21374_MOESM1_ESM.pdf]
